# Posterior Reversible Encephalopathy Syndrome Complicated by Aneurysm Interventional Embolization: A Case Report

**DOI:** 10.2174/0115734056384561250418065851

**Published:** 2025-04-28

**Authors:** Yi-Xuan Wang, Yang Liu, Jian-Feng Xu, Biao Jin

**Affiliations:** 1 Department of Neurosurgery, Affiliated Hospital of Southwest Medical University, Luzhou City, 646000, China; 2 Department of Neurosurgery, The Third Hospital of MianYang, MianYang, 621000, China

**Keywords:** Intracranial aneurysm, Status epilepticus, Posterior reversible encephalopathy syndrome (PRES), Cerebral vasospasm, Aneurysm embolization

## Abstract

**Introduction:**

Complications of Post-Reversible Encephalopathy Syndrome (PRES) following interventional embolization of aneurysms are rarely reported, and PRES disease can be reduced or resolved through prompt and aggressive treatment, resulting in minimal or no residual neurological deficits.

**Case Presentation:**

A 51-year-old female patient with an aneurysm in the pericallosal segment of the left anterior cerebral artery experienced prolonged status epilepticus following aneurysm embolization, attributed to PRES. The diagnosis of PRES was confirmed by symptom improvement and resolution of lesions on imaging studies after one month of treatment involving blood pressure management and prevention of cerebral vasospasm. At the 7-month post-discharge follow-up, the patient's examination indexes were normal without any residual neurological deficits.

**Conclusion:**

This case underscores the importance of promptly identifying and diagnosing PRES, as timely intervention can prevent permanent neurological deficits and mitigate the risk of more severe outcomes.

## 1 INTRODUCTION

Posterior Reversible Encephalopathy Syndrome (PRES) represents a distinctive pattern of vasogenic edema with a vascular origin that occurs in the setting of neurotoxicity. The documented occurrences of PRES commonly arise in conditions, such as malignant hypertension, renal insufficiency, eclampsia during pregnancy, administration of immunosuppressive or cytotoxic agents, autoimmune disorders, alcohol abuse, and organ transplants [1]. The prevailing hypothesis proposes that the rapid elevation of blood pressure leads to dysregulation of cerebral autoregulation and disruption of the blood-brain barrier, resulting in localized vasogenic edema. Alternatively, the vasospasm theory suggests that vasoconstriction due to a sudden surge in blood pressure or toxic agents triggers ischemia, cytotoxicity, and subsequent vasogenic edema in brain tissue [2]. Additionally, there are hypotheses, such as endothelial cell damage [3], attributing endothelial dysfunction to circulating endogenous or exogenous toxins. However, none of these theories comprehensively elucidate the etiology or pathophysiology of PRES, rendering the pathogenesis a subject of ongoing debate and controversy.

Common clinical manifestations of PRES include headache, nausea, vomiting, seizures, impaired consciousness, visual disturbances (including hemianopsia, cortical blindness, visual neglect, and decreased visual acuity), agitation, psychiatric abnormalities, unresponsiveness, and memory loss [4]. Intracranial aneurysms themselves can manifest with symptoms, such as headache and epilepsy, with some patients experiencing epileptic symptoms preoperatively. Compression of the cortex by the aneurysm may result in localized neuroglial cell proliferation, predisposing individuals with epilepsy related to cortical hyperplasia to an increased risk of postoperative seizures [5]. In a retrospective analysis of 24 documented cases showcasing transient neurological symptoms and head image anomalies following cerebral angiography akin to the current presentation, a significant proportion (71%) originated primarily from the neurosurgical domain. Among these cases, thirteen individuals (54%) exhibited abnormalities identified on Magnetic Resonance Imaging (MRI), displaying imaging characteristics indicative of PRES.

In 12 cases (50%), clinical manifestations included visual impairment, while altered mental status was observed in another 12 cases (50%). Additionally, altered mental status complicated by epilepsy was reported in only 2 cases (8%) [ 6 ].

Notably, both patients belonged to the subgroup of individuals with ruptured aneurysms who underwent cerebrovascular interventional embolization, presenting with additional comorbidities and lesions, such as subarachnoid hemorrhage and fluctuations in blood pressure. PRES is likely to be misdiagnosed or underdiagnosed because of the paucity of reports of PRES after vascular intervention in patients with intracranial aneurysms. In cases where a patient with an intracranial aneurysm develops sudden and persistent status epilepticus postoperatively, progressing to a comatose state, a high index of suspicion should be maintained for PRES. The presence of persistent status epilepticus could serve as an early indicator of PRES, underscoring the importance for clinicians to promptly initiate EEG in suspected PRES cases. Clinical presentations of PRES exhibit a range of manifestations, with generalized seizures being a prevalent feature, often accompanied by the potential for coma, as observed in our case. Prompt and intensive treatment can ameliorate or resolve symptoms. However, delayed diagnosis and intervention may result in progressive disease advancement, leading to irreversible neurological impairment and potentially fatal outcomes.

## 2 CASE REPORT

**Fig. (1):**
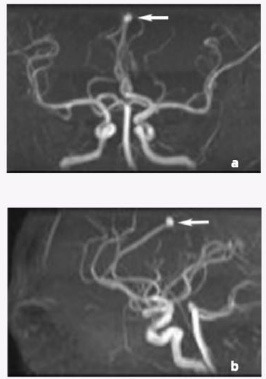
CT angiography of the patient on admission. (**a**, **b**) CT angiography shows an aneurysm (arrow) in the left cerebral artery at location A4.

The case is of a 51-year-old female with a history of hypertension, who presented with normal blood pressure at the time of admission. Outpatient CT angiography revealed an aneurysm of the left cerebral artery at location A4 (Fig. **1**).

**Fig. (2):**
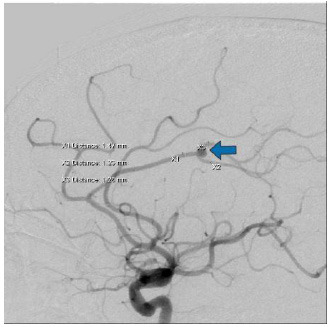
Cerebral angiogram of a patient just admitted to the hospital DSA shows a 5.5*4.0 mm aneurysm (arrow) in the pericallosal segment of the left anterior cerebral artery.

The neurologic examination at admission was unremarkable, and the initial test results did not show significant abnormalities. On the third day of admission, cerebral angiography (DSA) reconfirmed the presence of a 5.5*4.0 mm aneurysm at the same location, necessitating surgical intervention (Fig. **2**). After obtaining the patient's consent, oral anticoagulant therapy was initiated three days preoperatively (aspirin enteric-coated tablets 100 mg/day and clopidogrel 75 mg/day) to mitigate the risk of postoperative cerebral infarction. Intraoperatively, the aneurysm neck was treated with a flow diversion technique, and angiographic assessment revealed secure adherence to the vascular wall.

**Fig. (3):**
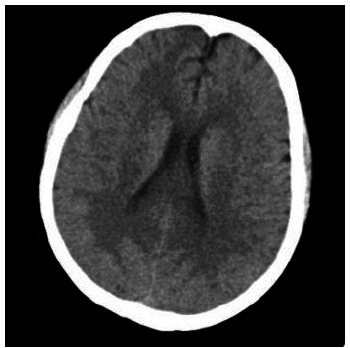
CT brain imaging on the day of onset Cranial CT examination suggested bilateral cerebral hemispheric symmetry, normal gray and white matter contrast, absence of abnormal density in the brain parenchyma, and no hemorrhage, infarction, or other new lesions.

Intraoperative blood pressure was maintained at 120/80 mmHg; however, 4 hours postoperatively, the patient experienced an acute generalized tonic-clonic seizure accompanied by altered consciousness and deviation of gaze towards the right. The seizure was transiently alleviated with an anticonvulsant (10 mg of diazepam) and followed by the administration of continuous sodium valproate. Thirty minutes later, the same symptoms reappeared, prompting a head CT scan that did not show evidence of hemorrhage, infarction, or new lesions (Fig. **3**).

**Fig. (4):**
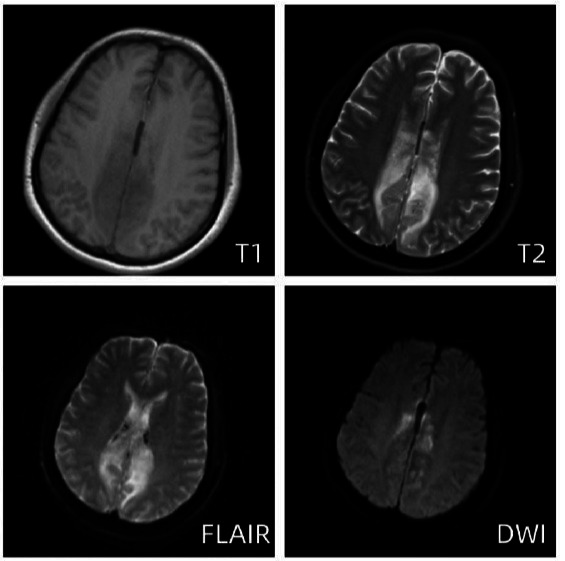
Brain MRI on day 5 after the onset of the disease. MRI showed that the T2 and FLAIR sequences of bilateral frontal-parietal paracentral and corpus callosum pressures showed markedly increased signal intensity in the cortex and subcortical white matter, suggesting vasogenic edema. DWI showed diffusion-restricted foci of bilateral parietal and corpus callosum pressures, suggestive of acute infarctions, while the remainder of the brain parenchyma exhibited no discernible abnormal signal patterns.

**Fig. (5):**
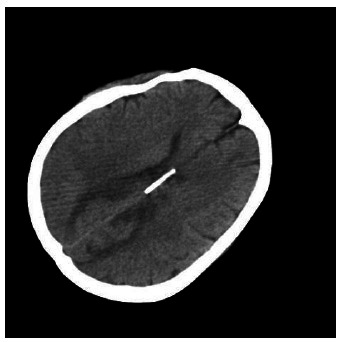
CT brain imaging on day 5 after onset of illness. CT shows striated hyperdense shadows in the region of the pericallosal segment of the anterior cerebral artery, along with newly observed hypodense areas with poorly defined borders in the parietal lobe proximal to the midline bilaterally and in the area of corpus callosum compression.

Subsequently, the patient continued to experience involuntary shaking of the right limb, with complex partial seizures propagating from the upper right limb to involve the entire body on multiple occasions, indicative of status epilepticus. Consequently, the patient was transferred to the Intensive Care Unit (ICU) for further evaluation and treatment. Serial transcranial Doppler (TCD) evaluations revealed reduced blood flow velocities in the bilateral middle cerebral arteries, bilateral internal carotid arteries, and bilateral vertebral arteries. Treatment measures included continuous administration of nimodipine to alleviate cerebral vasospasm and enhance cerebral perfusion, as well as mannitol to mitigate cerebral edema. On the fifth day in the ICU, magnetic resonance imaging demonstrated predominantly vasogenic edema in the parietal lobes and bilateral corpus callosum, characterized by symmetrically distributed hyperintense lesions on FLAIR (Fluid-Attenuated Inversion Recovery) imaging along with patchy and limited diffuse lesions on diffusion-weighted sequences (Fig. **4**). Concurrently, an emergent head CT showed new areas of hypodensity in the parietal lobe near the midline and corpus callosum bilaterally (Fig. **5**).

**Fig. (6):**
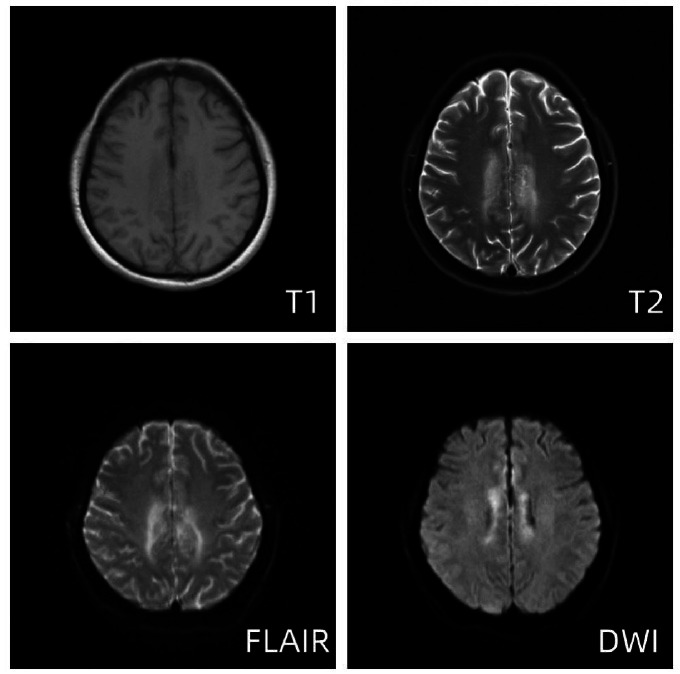
MRI of the brain on day 20 after onset of the disease. MRI results indicated slightly decreased signal intensity on T1-weighted images, with small bands of high signal observed near the midline of the bilateral frontoparietal lobes (specifically in the paracentral lobule region) on T2 and FLAIR sequences. DWI revealed restricted diffusion areas in bands adjacent to the midline of the bilateral frontoparietal lobes, showing a notable reduction in size compared to previous scans. The remaining brain parenchyma did not exhibit any conspicuous abnormal signal patterns.

A brain CT perfusion scan demonstrated reduced perfusion in the brain regions affected by vasogenic edema. Besides, dynamic EEG findings persisted, demonstrating epileptiform wave patterns. These combined findings prompted consideration of Reversible Encephalopathy Syndrome (PRES). In line with the provisional PRES diagnosis, treatment was initiated with edaravone and methylprednisolone, alongside stringent blood pressure management to optimize cerebral perfusion. Cerebral cryoprotection was employed to reduce cerebral metabolic demands, and the patient's regimen of antiepileptic medications and nimodipine was maintained. After a 12-day ICU stay, the patient was transferred to the neurosurgical unit upon recovery of consciousness and restoration of voluntary limb movements. As the patient's symptoms resolved, a corresponding reduction in white matter lesions was observed in imaging studies. On the 32nd day of hospitalization, a follow-up MRI revealed a significant reduction in vasogenic edema and improved perfusion of the lesion area compared with the previous one (Fig. **6**).

**Fig. (7):**
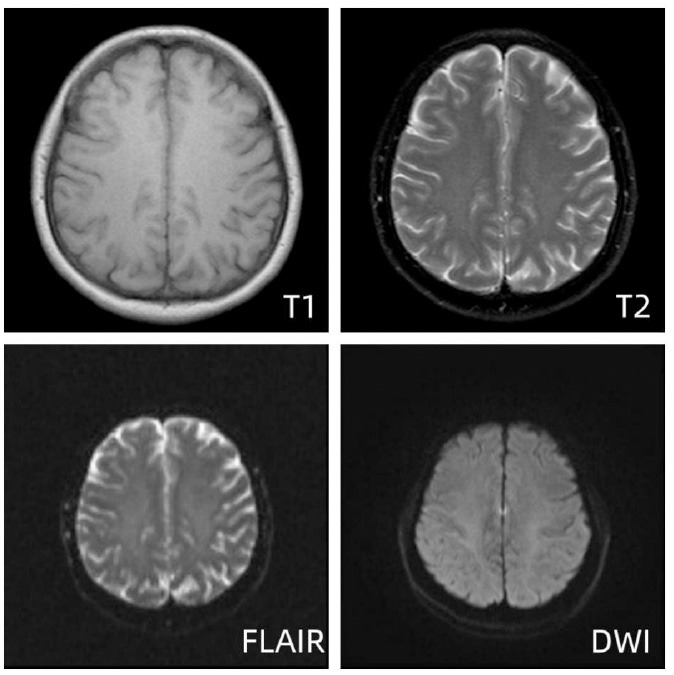
Follow-up MRI of the patient 7 months after discharge from the hospital MRI showed no significant abnormal signals within the brain parenchyma.

By one month post-operation, the patient exhibited full consciousness, alertness, orientation, and normal language and speech function. At the 7-month follow-up after discharge, Repeat Digital Subtraction Angiography (DSA) showed the intact placement of the stent, Electroencephalogram (EEG) suggested normal background activity without seizure patterns, and MRI imaging displayed complete normalization (Fig. **7**). Clinical assessments detected no signs of neurological deficits in the patient.

## 3 DISCUSSION

Endovascular interventional embolization is a minimally invasive procedure for the treatment of intracranial aneurysms, offering several advantages over conventional open aneurysm clamping. However, hemodynamic changes associated with this procedure can predispose some patients to postoperative complications, such as Cerebral Vasospasm (CVS) [7]. CVS manifests as intracranial vascular smooth muscle contraction, vessel diameter reduction, and localized transient ischemia at the blood supply site, which may further trigger neurological damage and severely affect the patient's prognosis [8]. The established mechanisms underlying the development of CVS encompass several factors, including dysregulation between vasoconstrictor and vasodilator agents, structural and functional abnormalities of the vascular wall, release of vasoactive compounds like angiotensin and catecholamines, immune-inflammatory reactions, perturbations in the equilibrium of free radical generation and elimination, and endothelial cell apoptosis [9].

After analysis, it was found that the application of contrast agents may contribute to the development of PRES. The pathogenesis involves two main mechanisms: First, the local injection of contrast medium into the brain elevates blood circulation system pressure, disrupting the autoregulation of cerebral arteries. This induces vasodilation in the vertebrobasilar system, leading to increased cerebral blood flow and hyperperfusion. Consequently, the integrity of the blood-brain barrier is disrupted, resulting in blood extravasation, hemorrhage, and edema.

Second, the contrast medium, being a hypertonic solution, can cause damage to vascular endothelial cells when administered in large quantities over a short duration [10].

In the diagnostic evaluation of RPES, computed tomography (CT) is only a preliminary method for detecting low-density lesions in posterior encephalopathy. However, Magnetic Resonance Imaging (MRI), particularly Fluid-attenuated Inversion Recovery (FLAIR) sequences, emerges as a superior diagnostic technique, offering heightened sensitivity in the detection of cortical and subcortical edema marked by more prominent high-intensity signal alterations than conventional sequences [11]. CT often suggests multiple patchy areas of low density in the bilateral fronto-parieto-occipital lobes, indicative of abnormal vasoconstriction in the brain among patients with PRES. This vasoconstriction leads to diminished cerebral blood flow, resulting in brain hypoxia and the emergence of abnormal signal intensities, such as low-density foci [12]. Upon MRI, white matter edema is characterized by high-signal T2 and low-signal T1 signals, typically symmetrically distributed in the posterior brain region [1]. These abnormal signal changes are reversible and tend to resolve as the patient's condition improves. The predominant neuroimaging findings in PRES typically manifest as focal areas of bilateral edema within the cerebral hemispheres, with a predilection for involvement of the parietal and occipital lobes, followed by the frontal lobes, temporo-occipital junction, thalamus, cerebellum, and spinal cord [13]. The vulnerability of the posterior circulation white matter to PRES is attributed to its high vascularity, sparse organization, and limited sympathetic innervation [14].

Various etiologies contribute to the pathogenesis of PRES, with the classical hypothesis proposing that severe hypertension disrupts autoregulatory mechanisms, leading to hyperperfusion, consequent endothelial injury, and the development of vasogenic edema [15]. Nonetheless, the patient in this case did not show a significant increase in blood pressure. Despite the absence of significant hypertension, suspicion of PRES should still be maintained, as research indicates that approximately 50% of PRES cases occur without pronounced elevations in blood pressure before symptom onset [16]. Various underlying conditions, medications, and medical circumstances that can induce blood pressure fluctuations, such as surgical interventions, should also be considered as potential risk factors for PRES.

Moreover, PRES triggers included hypertension, preeclampsia/eclampsia, renal failure, cytotoxic drugs and autoimmune diseases, and administration of immunosup-pressive drugs. Through a detailed inquiry of the patient's past history, no medications linked to renal dysfunction or cytotoxic effects were identified, and there was no prior exposure to immunosuppressants. The patient adhered to a regimen of antihypertensive therapy, maintaining well-controlled blood pressure levels. In summary, our analysis suggests that the primary contributors to PRES were the administration of contrast agents and cerebral vasospasm and inflammation induced by cerebrovascular interventions. Upon examining the case, the rapid local injection of hypertonic contrast media resulted in brain tissue hyperperfusion and vascular endothelial injury. This led to the extravasation of water molecules and macromolecules into the interstitium of brain cells through the compromised vascular endothelium. Damage to the blood-brain barrier and endothelial cells leads to endothelial dysfunction and the release of vasoactive substances, initiating vasospasm and subsequent tissue hypoxia. This cascade exacerbates the blood-brain barrier and endothelial damage, culminating in vasogenic cerebral edema. Cerebral vasospasm caused by cerebral tissue ischemia and hypoxia can disrupt brain cell metabolism, reducing ATP (Adenosine Triphosphate) production. Consequently, ATP-dependent sodium-potassium ion pumps malfunction, leading to intracellular sodium retention, cellular swelling, and the development of cytotoxic cerebral edema. Relief of cerebral vasospasm may trigger additional injury pathways. Ischemia-reperfusion injury can lead to an increase in free radicals, activation of the inflammatory response, calcium overload, and disruption of the blood-brain barrier. Based on the aforementioned analysis, early diagnosis of this condition is crucial. Treatment primarily focuses on addressing the underlying cause, including antiepileptic therapy, blood pressure regulation, alleviation of cerebral vasospasm, vasodilation, anti-inflammatory measures, and neutralization of free radicals. Additionally, interventions, such as mannitol and furosemide, may be employed to reduce intracranial pressure alongside other supportive symptomatic approaches as necessary. Supportive symptomatic treatments are also integral components of the management strategy for PRES. In this case, the patient received diazepam for symptom control and continuous pumping of sodium valproate, with midazolam added where appropriate, along with airway management. Midazolam is the preferred treatment for epilepsy due to its water solubility, non-irritating properties, rapid onset of action, and prompt penetration into the central nervous system following intravenous administration. It acts by enhancing the inhibitory transmitter γ- aminobutyric acid (GABA) receptor, facilitating chloride influx that leads to neuronal hyper polarization. This mechanism reduces neuronal excitability, suppresses abnormal neuronal discharges, terminates refractory epileptic seizures, and mitigates cerebral neurological damage [17]. Nimodipine, characterized by its high lipid solubility and rapid onset of action, exerts its therapeutic effect by reducing calcium influx, thus swiftly alleviating cerebral vasospasm in patients [18]. Studies have indicated significant clinical efficacy in treating cerebral vasospasm post-cerebral aneurysm with the combined administration of edaravone and nimodipine [19]. Edaravone, a novel free radical scavenger, functions as a potent small molecule hydroxyl radical scavenger and as an antioxidant. With a blood-brain barrier penetration rate of approximately 60%, edaravone demonstrates efficacy in scavenging cytotoxic hydroxyl radicals in the brain after ischemia/reperfusion. It attenuates delayed neuronal demise in peripheral ischemic hemianopsia subsequent to cerebral hemorrhage, inhibits lipid peroxidation, mitigates blood-brain barrier disruption, and ameliorates energy production impairments. By suppressing the generation of inflammatory mediators, such as leukotrienes, edaravone exerts anti-inflammatory effects, safeguards vascular endothelial cells, inhibits delayed neuronal cell demise, and alleviates cerebral edema [20]. Hormones have been shown to effectively diminish cerebral edema by safeguarding the integrity of the blood-brain barrier, reducing capillary permeability, counteracting inflammatory mediators, suppressing cytokine release, antagonizing oxygen free radicals, preserving cell membrane and lysosomal integrity, enhancing cerebral circulation and blood supply, maintaining sodium and calcium pump function, decreasing cerebrospinal fluid secretion, and aiding in inflammation resolution and cerebral edema reduction when administered judiciously. In this case, the administration of methylprednisolone yielded satisfactory treatment outcomes. Mannitol was administered to address cerebral edema, furosemide was employed when required, and strict monitoring and control of the patient's fluid balance were implemented. Due to the lack of sufficient literature documenting cases of PRES complicated by embolization of unruptured aneurysms presenting with coma and recurrent epileptic seizures as predominant clinical features, standardized treatment guidelines for this scenario remain unknown.

Epidemiologic data suggest that PRES can affect individuals across all age groups, with a higher prevalence observed in females than males [21]. Clinical and imaging features of PRES in children are comparable to those seen in adults. Evidence suggests that, alongside vasogenic edema, cytotoxic edema may also be present, particularly in cases where timely intervention is lacking, underscoring the potential for irreversible damage associated with PRES [22]. Early recognition and prompt implementation of comprehensive etiological and symptomatic treatments are crucial in the management of PRES.

## 4 PATIENT PERSPECTIVE

The patient was satisfied with the treatment and she said she was discharged from the hospital without any after-effects of this treatment.

## CONCLUSION

In conclusion, cerebral vasospasm continues to be a prevalent complication following interventional embolization of aneurysms. Despite this, limited knowledge exists regarding the co-occurrence of PRES following intracranial aneurysm embolization, potentially resulting in an underestimated incidence and underdiagnosis within this treatment modality. This case stands out as a unique illustration of successful early diagnosis and management of a patient presenting with PRES complicated by interventional embolization of an unruptured aneurysm. Prompt emergency cranial CT was conducted upon the initial onset of altered clinical status to assess for the presence of any new lesions. After excluding new lesions, the patient received symptomatic care. In instances where symptoms persisted or worsened, particularly if accompanied by significant mental status alterations, the patient underwent proactive airway management and was transferred to the intensive care unit. Subsequent cranial imaging was utilized to monitor and confirm the diagnosis of PRES. In cases where severe mental status changes complicate epilepsy following intervention for unruptured aneurysms, prompt and appropriate supportive care and symptomatic treatments play a crucial role. These interventions may include antiepileptic therapy, blood pressure regulation, alleviation of cerebral vasospasm, vasodilation, anti-inflammatory measures, free radical scavenging, and reduction of intracranial pressure through dehydration with mannitol and furosemide as warranted.

The purpose of this case report is to enhance physicians' comprehension of this condition, enabling improved recognition and management and ultimately contributing to enhanced patient outcomes. The prospective accumulation of similar cases holds promise for advancing our understanding of the pathophysiology of PRES and for developing a more comprehensive and nuanced treatment regimen.

## Data Availability

Not applicable.

## LIST OF ABBREVIATIONS

PRES: Posterior Reversible Encephalopathy Syndrome
CVS: Cerebral vasospasm
CT: Computed tomography
MRI: Magnetic resonance imaging
FLAIR: Fluid-attenuated inversion recovery
DWI: Diffusion Weighted Imaging
ICU: Intensive care unit
TCD: Transcranial Doppler Ultrasound
ATP: Adenosine triphosphate
DSA: Digital subtraction angiography
EEG: Electroencephalogram
GABA: γ- aminobutyric acid

## STANDARDS OF REPORTING

CARE guidelines were followed.
